# Anthropogenic or Natural Dispersal: Case of the Spiny‐Tailed Iguanas ( *Ctenosaura* ) on Clarion Island, Mexico

**DOI:** 10.1002/ece3.72366

**Published:** 2025-10-24

**Authors:** Daniel G. Mulcahy, Jacobo Reyes‐Velasco, Daniel I. Vázquez‐Arce, Juan A. Cervantes‐Pasqualli, Juan E. Martínez‐Gómez, Kevin de Queiroz

**Affiliations:** ^1^ Museum für Naturkunde, Leibniz Institute for Evolution and Biodiversity Science Berlin Germany; ^2^ National Museum of Natural History Washington DC USA; ^3^ Herp.Mx A.C Colima Mexico; ^4^ Instituto de Ecología, AC Red de Interacciones Multitróficas Veracruz Mexico

**Keywords:** divergence times, herpetofauna, island conservation, mtDNA, Pacific Ocean, Revillagigedo Islands

## Abstract

Clarion Island, in the Revillagigedo Archipelago off the Pacific Coast of Mexico, hosts a unique assemblage of vertebrates. Introduced species have caused significant ecological damage, and Spiny‐tailed Iguanas ( *Ctenosaura pectinata* ) were assumed to have been introduced in recent times, prompting plans for eradication. To investigate the origin of the *Ctenosaura* population on Clarion Island, we conducted phylogenetic analyses of the Clarion Island and mainland populations using a portion of the mitochondrial DNA gene ND4. We estimated the date of divergence of the Clarion Island population from mainland Mexico populations using a relaxed‐clock method. Phylogenetic analyses reveal that Clarion iguanas are sister to mainland *C. brachylopha* populations in northwestern Mexico, a species recently resurrected out of *C. pectinata* . We estimated a divergence of approximately 425,600 years ago for the Clarion population—predating human colonization of the Americas. These findings support natural dispersal, likely through rafting on vegetation mats, as the mechanism of colonization. Iguanas are well known for their ability to colonize islands, and this represents their second‐longest overwater dispersal (> 1100 km); slightly greater than the distance of the Galapagos Islands from mainland Ecuador. Our findings demonstrate that Spiny‐tailed Iguanas are native to Clarion Island and should be considered an integral part of the island's native fauna. Conservation plans must prioritize the protection of this population, which we identify as an evolutionarily significant unit (ESU). Further genetic sampling and analyses are needed to determine the population's genetic variation and taxonomic status. Our findings challenge prior assumptions and emphasize the need for evidence‐based conservation strategies to preserve the integrity of oceanic island ecosystems.

## Graphical abstract

Species can colonize oceanic islands by both natural and human‐assisted dispersal and human‐caused species introductions have had catastrophic effects on ecosystems around the world. The Spiny‐tailed Iguanas inhabiting Clarion Island were previously considered introduced. Molecular data and analyses support iguanas' arrival on Clarion Island by natural dispersal long before humans were in the Americas.

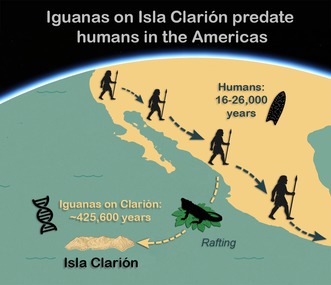

## RESUMEN

La Isla Clarión, en el archipiélago de Revillagigedo, frente a la costa del Pacífico mexicano, alberga un conjunto único de vertebrados. Las especies introducidas han causado importantes daños ecológicos, y se cree que las iguanas de cola espinosa (*Ctenosaura pectinata*) han sido introducidas recientemente, lo que ha dado lugar a planes de erradicación. Para investigar el origen de la población de *Ctenosaura* en la Isla Clarión, realizamos análisis filogenéticos de las poblaciones de *Ctenosaura* de la Isla Clarión y de México continental utilizando una porción del gen ND4 del ADN mitocondrial. Estimamos la fecha de divergencia de la población de la Isla Clarión respecto a las poblaciones de México continental utilizando un método de reloj relajado. Los análisis filogenéticos revelan que las *Ctenosaura* de Clarión son el grupo hermano de las poblaciones continentales de *C. brachylopha* en el noroeste de México, una especie recientemente resucitada de *C. pectinata*. Estimamos una divergencia de hace aproximadamente 425.600 años para la población de Clarión, antes de la colonización humana de las Américas. Estos resultados sugieren dispersión natural, probablemente a través de balsas de vegetación, como mecanismo de colonización. Iguanas del género *Ctenosaura* son bien conocidas por su capacidad para colonizar islas y ésta representa la segunda mayor dispersión sobre el agua (> 1.100 km) de cualquier iguana; ligeramente mayor que la distancia de las Islas Galápagos desde el Ecuador continental. Nuestros hallazgos demuestran que las iguanas de cola espinosa son nativas de la Isla Clarión y deben considerarse parte integrante de la fauna autóctona de la isla. Los planes de conservación deben priorizar la protección de esta población, que identificamos como una unidad evolutivamente significativa (ESU). Son necesarios más muestreos y análisis genéticos para determinar la variación genética de la población y su estatus taxonómico. Nuestros hallazgos desafían las suposiciones previas y enfatizan la necesidad de estrategias de conservación basadas en la evidencia para preservar la integridad de los ecosistemas insulares oceánicos.

## 1 Introduction

Human activities have facilitated the global movement of microbes, plants, and animals, either intentionally or unintentionally, for centuries (e.g., “portmanteau biota” brought by Europeans to the Americas; Crosby 1986). Such introductions have often had devastating effects on native ecosystems, especially when organisms become established in non‐native habitats. Amphibians and reptiles are no exception, with numerous examples of introduced species severely impacting local fauna. For instance, Cane Toads ( *Rhinella marina* ) in Australia have caused widespread ecological damage (Lever 2001; Shine 2014; Jolly et al. 2015). Islands are particularly vulnerable to invasive species due to their isolated ecosystems and limited native biodiversity, as seen with Brown Tree Snakes ( *Boiga irregularis* ) in Guam (Rodda et al. 1992; Wiles et al. 2003).

Iguanas (Buckley et al. 2016) are notable examples of reptiles capable of both human‐mediated (e.g., Avery et al. 2014) and natural dispersal to islands (e.g., Rassmann 1997; Scarpetta et al. 2025). They are often introduced intentionally as a food source or unintentionally as stowaways during transport (Davy et al. 2011; Knapp et al. 2021). Populations established from these introductions can profoundly alter island ecosystems, competing with native species and modifying habitats. Clarion Island, the westernmost island in the Revillagigedo Archipelago, Colima, Mexico, presents an intriguing case of purportedly introduced iguanas (Martínez‐Gómez 2003; Aguirre‐León and Matías‐Ferrer 2017). This small island (~19.8 km^2^), located 1098 km west of Manzanillo, Colima, and 710 km southwest of Los Cabos, Baja California Sur, has a history of human impacts. Since the establishment of a Mexican naval outpost in 1979, the island has experienced the introduction of various non‐native species, including pigs, sheep, and rabbits, which significantly altered its native flora (Mendez‐Guardado 2001). While pigs and sheep were eradicated in the 2000s, rabbits remain abundant and continue to impact the island's vegetation (Aguirre‐Muñoz et al. 2011). Clarion Island is the only island in the archipelago with iguanas (González‐Sánchez et al. 2021).

Clarion Island is the oldest island (Pliocene) in the Revillagigedo Archipelago, a small group of seamount islands that rose independently along the Clarion Fracture Zone on the Pacific Plate extending west from the Rivera Plate, a microplate between the Pacific, North American, and Cocos plates situated between Baja California and Puerto Vallarta, Mexico (Bryan 1967; B. H. Brattstrom 1990; Gottscho 2016). Since the late 19th century, naturalists have explored Clarion Island, documenting its unique and often challenging environment formerly characterized by dense cactus thickets and tall grasses. Early expeditions, such as those of Townsend in 1889 (Townsend 1890), Webster‐Harris in 1897 (Rothschild and Hartert 1899), and the California Academy of Sciences in 1925 (Hanna 1926; Slevin 1926), laid the groundwork for understanding the island's biodiversity. Among their discoveries were endemic reptiles, such as Clarion Whipsnakes ( *Masticophis anthonyi* ) and Clarion Tree Lizards ( *Urosaurus clarionensis* ), as well as Clarion Nightsnakes ( *Hypsiglena unaocularus* ) (Tanner 1944), which were dismissed as a locality error (Brattstrom 1955) but later rediscovered (Mulcahy et al. 2014).

Two lizard species are currently considered introduced to the Revillagigedo Archipelago: Common House Geckos ( *Hemidactylus frenatus* ), first reported on Socorro Island (Galina‐Tessaro et al. 1999; Medina‐Salazar 2020), and Guerreran Spiny‐tailed Iguanas ( *Ctenosaura pectinata* ), which were thought to have been introduced to Clarion Island around 1994 (Martínez‐Gómez 2003), or perhaps earlier (1979–1991). Iguanas were thought to have been introduced by military personnel at least 25 years prior to the expedition (in 2016) “…shortly after the establishment of their garrison in 1979” (Scheidt 2017, 123). Unlike *Hemidactylus frenatus* , which is commonly associated with human settlements, the presence of *Ctenosaura pectinata* raises questions about its origin and mode of arrival. On one hand, given Spiny‐tailed Iguanas (*Ctenosaura*) are consumed as food in many parts of Mexico (Reynoso et al. 2020), their purposeful transport to such a remote location seems possible. On the other hand, other species of reptiles on Clarion Island are thought to have naturally dispersed over water, such as the tree lizards (Feldman et al. 2011), nightsnakes (Mulcahy et al. 2014), and whipsnakes (Myers et al. 2017).

**FIGURE 1:**
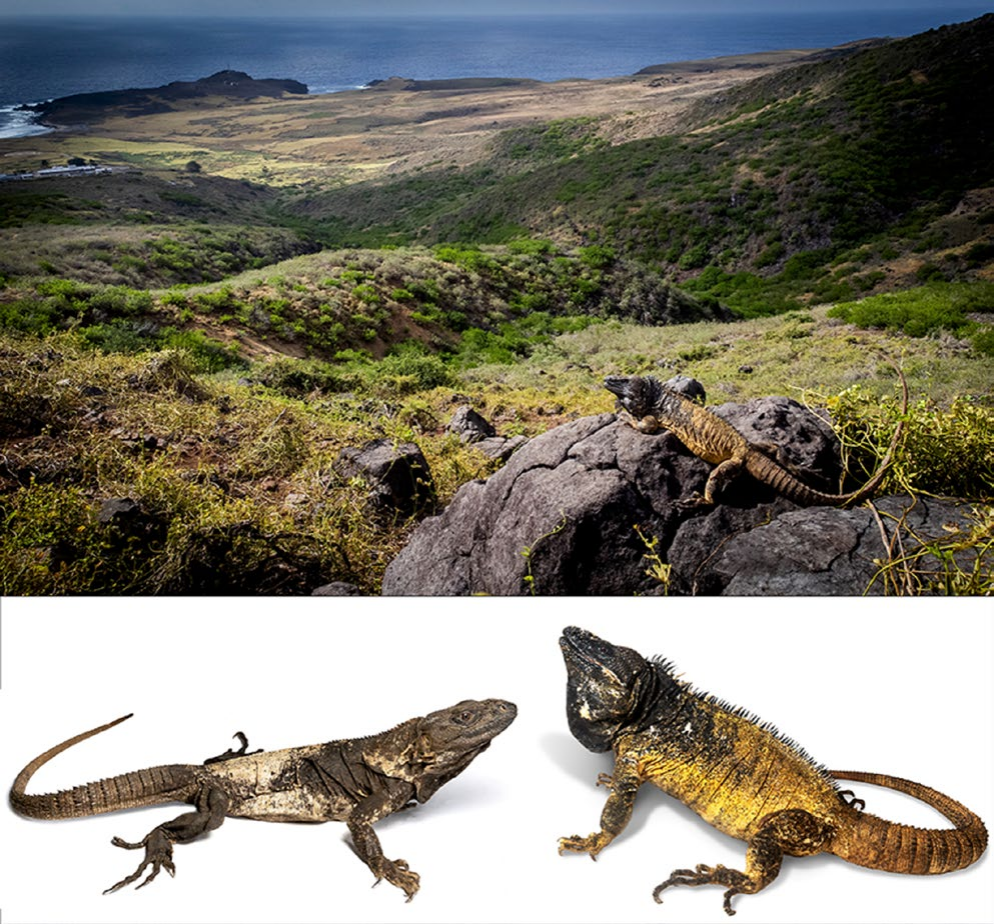
Top, in situ photograph of *Ctenosaura brachylopha* basking on Clarion Island in 2023. Bottom left, adult *C. pectinata* from Colima; bottom right, adult male *C. brachylopha* from Clarion Island. The naval base can be seen in the upper left. All images by Jacobo Reyes‐Velasco.

In this study, we investigate the origin of Spiny‐tailed Iguanas on Clarion Island (Figure 1). By analyzing mitochondrial DNA and morphological data, we compare members of the Clarion population with those of the *Ctenosaura pectinata* species complex along Mexico's western coast—including the recently resurrected *Ctenosaura brachylopha* (Zarza et al. 2019). Our findings support an earlier divergence for the Clarion population than can be explained by human‐mediated transport. These results provide critical insights into the biogeography of the Revillagigedo Archipelago's fauna and underscore the importance of understanding the origins of insular species for effective conservation and ecosystem management.

## 2 Materials and Methods

### 2.1 Sampling and Earlier Surveys

Three of us (D.G.M., J.E.M.‐G., and J.A.C.‐P.) visited Clarion Island from 19 May to 6 June 2013, with the intention of searching for Nightsnakes (*Hypsiglena*) (Mulcahy et al. 2014), though other reptiles, including birds, squamatans (snakes and lizards), and marine turtles were observed and recorded. Visual‐encounter surveys were conducted day and night over most of the island. J.E.M.G. and colleagues had previously visited the island regularly since 1988, primarily surveying birds, but also monitoring Clarion Whipsnakes ( *Masticophis anthonyi* ) and Clarion Tree Lizards ( *Urosaurus clarionensis* ). J.E.M.G. and colleagues noted the occurrence of iguanas (*Ctenosaura*), but assumed they were introduced since J.E.M.G.'s expedition in 1995 (Martínez‐Gómez 2003). Fieldwork was approved by the Dirección General de Vida Silvestre at the Secretaría de Medio Ambiente y Recursos Naturales (SEMARNAT), Comisión Nacional de Áreas Naturales Protegidas (CONANP), and Secretaría de Marina (SEMAR), and followed agency's regulations required for humane and ethical procedures for wildlife handling and obtaining permits (see Acknowledgments). Specimens of *Ctenosaura* were hand‐captured and one voucher specimen was collected, a subadult from near the naval base, which was euthanized with 20% benzocaine. A tissue sample was taken from the liver and stored in 95% EtOH, and the specimen was fixed in 10% formalin and deposited in the Colección Nacional de Anfibios y Reptiles (CNAR‐IBH 28173) at the Instituto de Biología, Universidad Nacional Autónoma de México.

### 2.2 Recent Visual Surveys and Morphology

**FIGURE 2:**
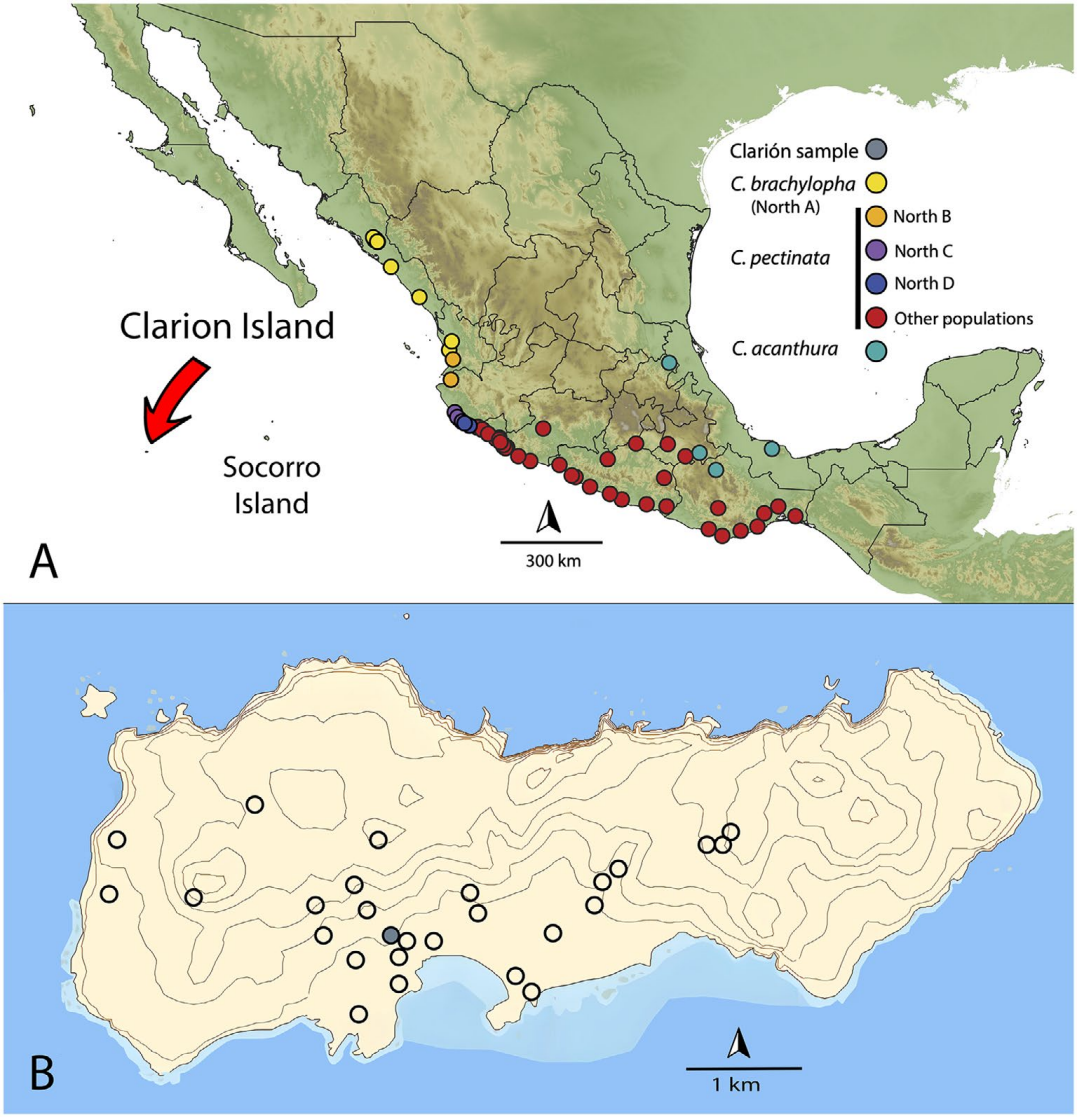
Map of Mexico including Clarion Island. (A) Sampling localities of Zarza et al. (2008, 2019) for the mitochondrial gene ND4. Yellow, orange, purple, and blue dots indicate membership in clades North A–D, respectively; red dots represent the remaining samples. The lightest yellow and northwestern‐most (from Sinaloa–Nayarit) dots represent samples identified as *C. brachylopha* by Zarza et al. (2019), the remaining dots refer to specimens identified as “ *Ctenosaura pectinata* ” and *C. acanthura* . (B) Map of Clarion Island showing location of observed iguanas in 2013 and 2023. Blue dot shows location of the naval base where the voucher specimen was taken. Contour lines represent 50‐m intervals.

Two of us (J.R.‐V. and D.I.V.‐A.) visited Clarion Island from November 6 to December 8, 2023, to document the reptiles of Clarion Island. This visit had the approval of the Secretaría de Gobernación (SEGOB), Secretaría de Marina (SEMAR), and the Comisión Nacional de Áreas Naturales Protegidas (CONANP) (see Acknowledgments). Visual surveys were carried out during the day and at night throughout most of the island. Reptiles were photographed whenever encountered and their locations registered (Figure 2); however, no voucher specimens were collected. Subsequently, one of us (JRV) visited the CNAR‐IBH and examined the voucher specimen from Clarion Island and other *Ctenosaura* specimens from western mainland Mexico for comparison of morphological characters distinguishing *C. brachylopha* from *C. pectinata* (Bailey 1928; Zarza et al. 2019).

### 2.3 Sequencing Protocols

Sequencing of DNA was conducted on the mitochondrial gene region ND4. Extractions of genomic DNA were performed on an Auto‐Genprep 965 (2011 AutoGen Inc.), using standard phenol manufacturer protocols. Genomic DNA was eluted in 100 μL of AutoGen R9 re‐suspension buffer. Polymerase chain reactions were performed in 10 μL reactions for mtDNA locus ND4–tRNA^His–Ser^ (hereafter referred to as “ND4 data”) using the primers HypNad4f1 and HypLeu2r1 and PCR thermocycler profile of Mulcahy (2008). The PCR reactions were run on agarose gels and purified with ExoSapIT enzyme. Cycle‐sequence reactions were performed in both directions using PCR primers and BigDye Terminator v3.1 Cycle‐Sequencing Kits in 10 μL reactions and run on an Automated ABI3730 Sequencer (2011 Life Technologies). Raw chromatograms were edited in Sequencher v5.1 (2012 Gene Codes Corp.), complementary strands were aligned, and protein encoding regions were inspected for translation. The sequence was queried in NCBI's GenBank nucleotide database using blastn searches for verification and deposited in GenBank (Accession number: PV941008).

### 2.4 Phylogenetic Analyses

Ninety‐six unique haplotypes of the mitochondrial DNA (mtDNA) locus ND4 of *Ctenosaura pectinata* , *C. acanthura*, and *C. brachylopha* (Figure 2A), and one each of the outgroups *C. hemilopha* (EU246705) and *C. similis* (EU246704), were downloaded from GenBank (EU246698–EU246780, Zarza et al. 2008; FJ715723–FJ715736, Zarza et al. 2011; KT003209–KT003210, Zarza et al. 2019). The sampling of Zarza et al. (2008, 2011, 2019) was much denser along the northwestern coast of Mexico than depicted here because we used only unique haplotypes, and only one locality for each is shown in Figure 2. These sequences are 561 bp in length, and (in contrast to our sample) do not include the tRNAs^His–Ser^ that follow ND4. Our sequence, 817 bp in length, was trimmed to the same length and aligned with the sequences from GenBank using the Muscle option in Geneious Prime (v2024.0.5). Uncorrected pairwise percent distances were calculated in Geneious Prime. The alignment was inspected for premature stop codons; none were detected. Maximum‐likelihood (ML) phylogenetic analyses were conducted using IQ‐Tree v1.6.12 (Nguyen et al. 2015), using ModelFinder to determine the best fit model (TN + F + G4) based on the Bayesian Information Criterion (Kalyaanamoorthy et al. 2017). This was followed by the best‐tree search and combined SH‐like approximate likelihood‐ratio test (SH‐aLRT; Guindon et al. 2010) and ultra‐fast bootstrap analyses (Hoang et al. 2018), each with 1000 replicates.

### 2.5 Divergence Dating

To test whether the origin of the Clarion *Ctenosaura* population predates the possibility of human transport, we estimated the date of divergence between the Clarion Island sample and its closest mainland relatives. We used the software package BEAST2 (v2.7.7; Bouckaert et al. 2019) using the calibration point and mutation rate previously estimated for the *C. pectinata* complex and the ND4 gene region (Zarza et al. 2008). We used the TN93 substitution model, the gamma site heterogeneity model with four rate categories, empirical base frequencies, and the optimized (uncorrelated) relaxed clock model (Douglas et al. 2021). We set the mean clock rate = 0.0078 but used SD = 0.6 (instead of SD = 0.0018 used by Zarza et al. 2008) to allow for greater rate variation among lineages, with the “mean in real space” option. We used the Calibrated Yule Model for the tree prior, with a log‐normal birth rate prior, and a calibration point (with a normal distribution) for the most recent common ancestor (mrca) of *C. hemilopha* , *C. pectinata* , and *C. acanthura* (mean = 9.24 Ma, SD = 2.47). This calibration point was estimated based on an earlier analysis using 12 species of extant iguanid lizards and two fossil calibrations, with upper and lower bounds of Iguaninae set to 93–24 Ma (Zarza et al. 2008). A more recent study, using 8 nuclear loci and 18 fossil calibrations, estimating dates of divergence among iguanian lizards resulted in a younger (4.8 Ma) estimate for this node (Malone et al. 2017). To accommodate this difference, we ran a similar analysis as listed above, with the exception of a younger calibration point (mean = 4.8 Ma and SD = 1.5) for the *C. hemilopha, C. pectinata*, and *C. acanthura* node (Malone et al. 2017). Analyses were run for 50 million generations, sampling every 10,000. The log file results were analyzed using Tracer (v1.7.2; Rambaut et al. 2018) to assess burn‐in values and check effective sample size (ESS) values, which should be above 200. TreeAnnotator (part of the BEAST2 software package) was used to generate a maximum clade credibility tree for the resulting 45,000 trees, with a 10% burn‐in period. We then used the resulting 95% highest posterior density (HPD) interval for the node uniting the Clarion sample and its closest mainland relatives to test if the divergence predates human presence in the region.

## 3 Results

### 3.1 Recent Visual Surveys and Morphology

**FIGURE 3:**
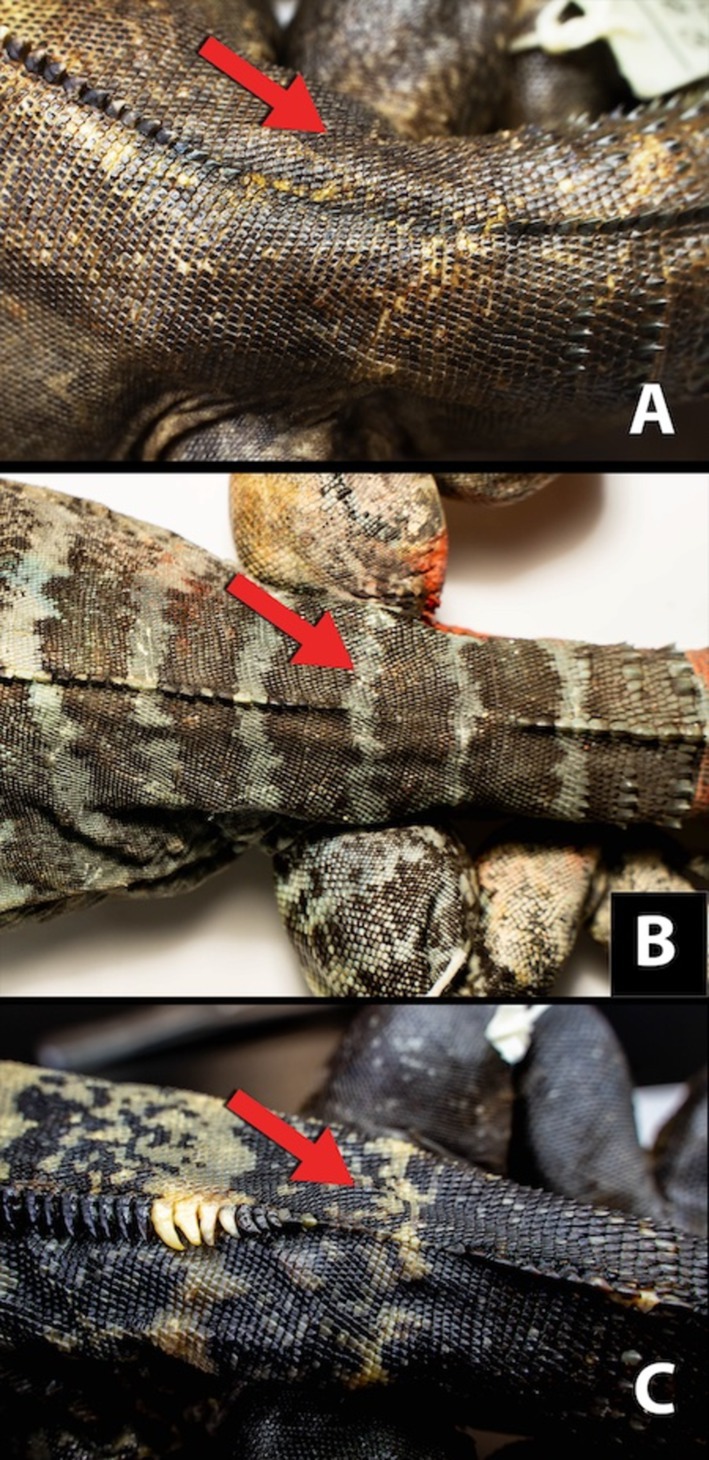
Comparison of enlarged mid‐dorsal scales in *Ctenosaura* species. The red arrow indicates the region above the pelvic girdle, a key characteristic for distinguishing between *C. brachylopha* and *C. pectinata* . In *C. brachylopha*, the mid‐dorsal row of enlarged scales is discontinuous above the pelvic girdle, whereas in *C. pectinata* , the row of enlarged scales is continuous. (A) *C. brachylopha* from near Culiacán, Sinaloa (CNAR−IBH 8275). (B) Individual from Clarión (CNAR−IBH 28173). (C) *C. pectinata* from Colima (CNAR−IBH 8276).

Between November and December 2023, we observed a total of 132 iguanas. However, because the individuals were not marked, some observations may represent repeat sightings of the same animals. Most of our time was spent near the naval base in the southern part of the island, where the majority of iguana sightings occurred (Figure 2B). Iguanas appeared particularly abundant in the steep, south‐facing canyons, where the largest males were observed. Due to the limited number of captures, we were unable to accurately determine the sex of most individuals. Juvenile iguanas were observed exclusively near the naval base, with only about 10 juveniles recorded. Iguanas were observed across most parts of the island, except for the eastern section, which was surveyed during only a single day. The specimen CNAR‐IBH 28173, collected during the 2013 expedition, has a dorsal crest that ends before the sacral region and the first seven whorls of spinous caudal scales are separated by three rows of small flat scales (Figure 3), matching the condition in *C. brachylopha* and distinguishing that species from *C. pectinata* (Bailey 1928; Zarza et al. 2019).

### 3.2 Phylogenetic Analyses

**FIGURE 4:**
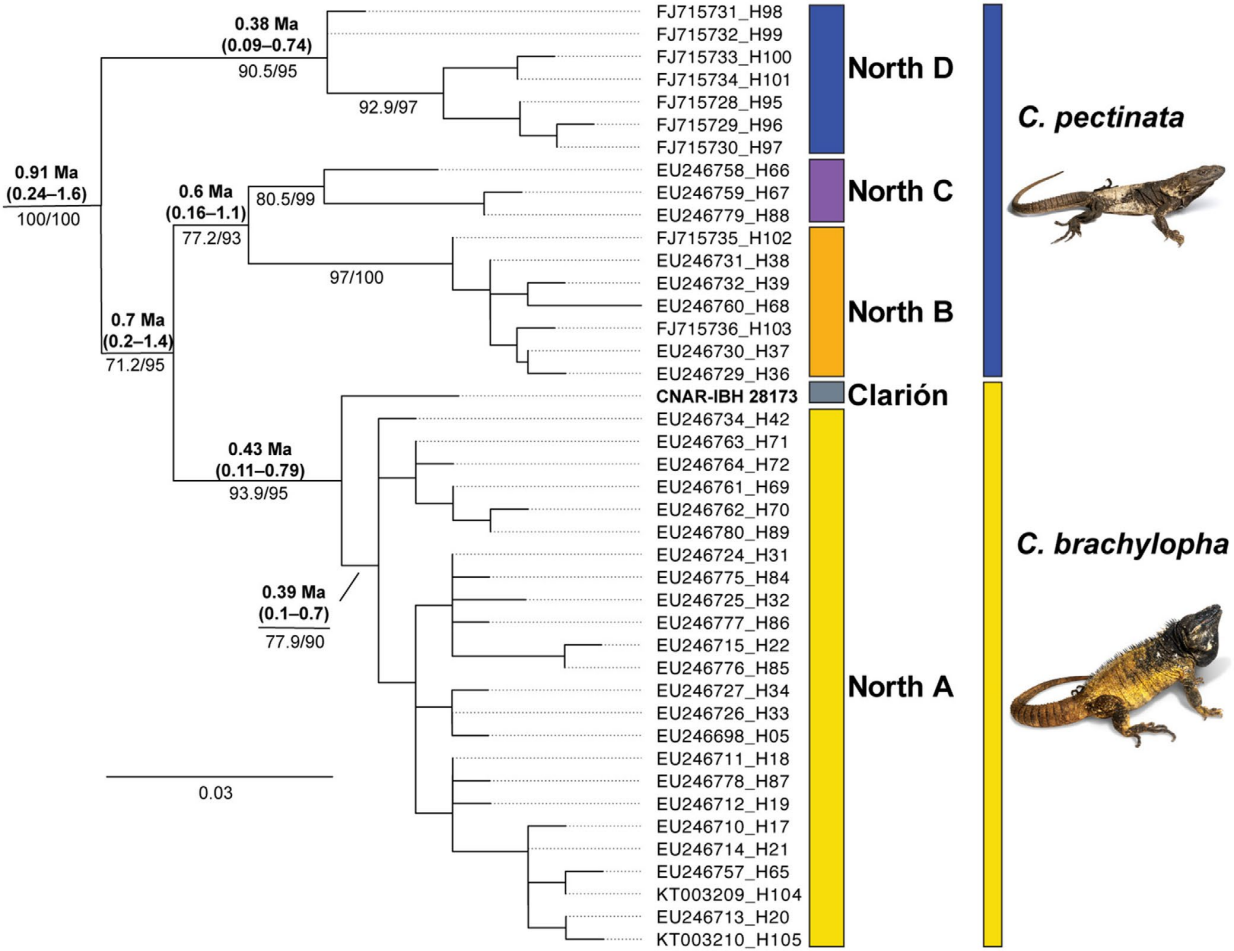
Maximum‐likelihood phylogeny based on 561 base pairs of ND4 for the northwestern part (clades North A–D) of the *Ctenosaura pectinata* species group. See Appendix 1 Figure A1 for full tree. Values at branches in regular font indicate the SH‐aLRT/ultrafast bootstrap support values for relevant nodes, and values in bold indicate mean values (Ma) and 95% HPD intervals from the Bayesian divergence dating analysis. The Clarion Island sample is indicated by voucher number (CNAR‐IBH 28173), while the other samples are labeled by GenBank accession and haplotype numbers as designated by Zarza et al. (2019). The scale bar indicates the expected number of substitutions per site.

**FIGURE A1:**
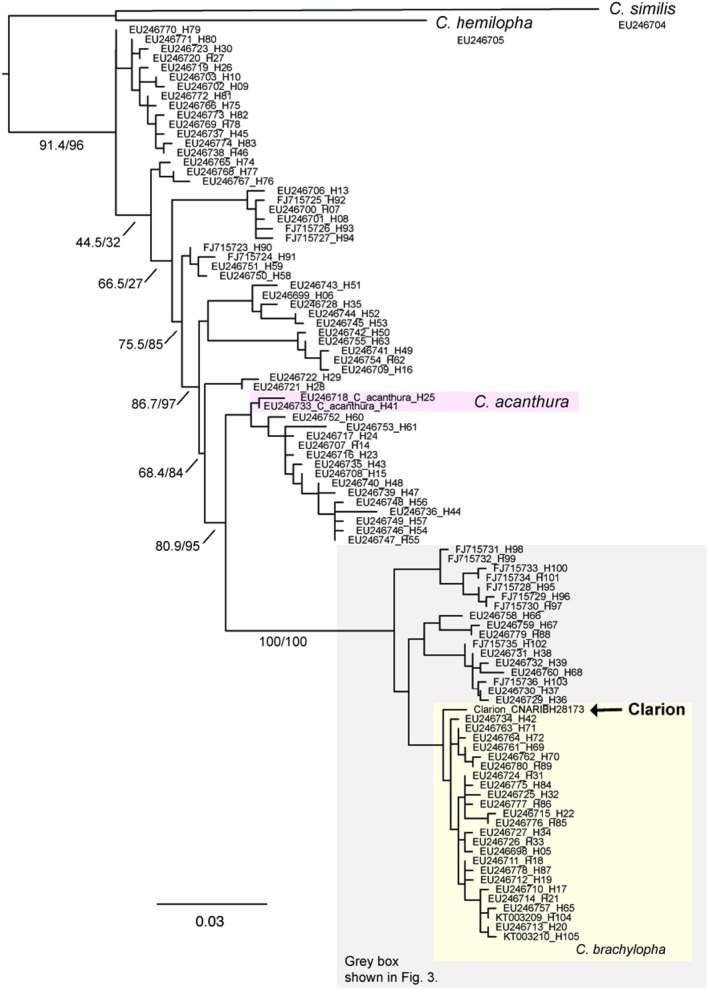
Maximum‐likelihood phylogeny of all ND4 haplotypes. Tip names are GenBank accession and haplotype numbers. *C. brachylopha* and *C. acanthura* are shown in yellow and pink boxes respectively, all other haplotypes represent *C. pectinata* (outgroups: *C. similis* and *C. hemilopha* ). Branches values indicate SH‐aLRT/ultrafast bootstrap support for relevant nodes.

Our maximum‐likelihood analyses produced a similar topology to those reported by Zarza et al. (2008, 2019). The Clarion Island sample (CNAR‐IBH 28173) was placed sister to the North A clade (*C. brachylopha*) of Zarza et al. (2019) with 93.5% SH‐aLRT and 95% ultrafast bootstrap support values (Figure 4; full tree in Appendix 1, Figure A1). The Clarion Island ND4 haplotype was found to be between 0.9% and 1.8% different from haplotypes in the North A clade and ranged from 1.8% to 3.4% different from haplotypes in clades North B–D (Table 1). The Bayesian relaxed‐clock analyses resulted in trees with similar topologies. Based on the analysis with the 9.24 Ma calibration, the mrca for the Clarion sample and North A clade was estimated to be 0.7653 Ma, with a 95% HPD interval of 0.2005–1.3833 Ma. The analysis based on the younger 4.8 Ma calibration point estimated the mrca for the Clarion sample and North A clade to be 0.4256 Ma, with a 95% HPD interval of 0.1126–0.7892 Ma (Figure 4).

**TABLE 1:** Minimum and maximum uncorrected percent differences for pairwise comparisons of ND4 between the haplotype of the Clarion specimen and those of clades North A–D.

Clade	North A	North B	North C	North D
CNAR‐IBH 28173	0.90–1.81	2.53–2.89	1.82–2.71	2.53–3.43
North A	0.18–1.43	2.14–3.21	2.14–3.74	1.96–3.74
North B		0.18–2.67	1.78–2.85	2.50–3.74
North C			0.18–1.07	2.50–3.74
North D				0.18–1.43

*Note:* Diagonal values are for comparisons between unique haplotypes within the four clades.

## 4 Discussion

The Revillagigedo Archipelago hosts a unique assemblage of reptiles, including two endemic lizard species ( *Urosaurus auriculatus* and *Urosaurus clarionensis* ), two endemic snake species ( *Hypsiglena unaocularus* and *Masticophis anthonyi* ), a widespread marine turtle species ( *Chelonia mydas* ), and at least 11 endemic bird species or subspecies (Brattrom and Howell 1956; Brattstrom 1990). Among these, Clarion Island harbors at least one endemic lizard species ( *U. clarionensis* ) and two endemic snake species ( *H. unaocularus* and *M. anthonyi* )—the highest number of any island in the archipelago. Clarion Island also has three endemic bird species or subspecies: Clarion Mourning Doves ( *Zenaida macroura clarionensis* ), Clarion Wrens ( *Troglodytes tanneri* ), and Townsend's Shearwaters (*Puffinus a. auricularis*); see Martínez‐Gómez et al. (2015) for the current taxonomic status of the latter. Clarion Ravens ( *Corvus corax clarionensis* ) were previously considered endemic by some (Brattstrom 1990), but they are now considered more widespread in western North America (Rea 1986; Omland et al. 2000). This high level of endemism underscores the importance of understanding the origins of the island's terrestrial fauna, including the Spiny‐tailed Iguanas, which is critical for management and conservation.

### 4.1 Natural Dispersal of *Ctenosaura* to Clarion Island

Our phylogenetic analyses reveal that the Clarion Island Spiny‐tailed Iguana sample is sister to all mainland *Ctenosaura brachylopha* samples in northwestern Mexico (Figure 4). The Clarion Island ND4 Haplotype was found to be 0.9%–1.8% divergent (Table 1) from those of the North A clade of Zarza et al. (2019). The younger (more conservative) of the two age estimates from our divergence dating analyses was 425,600 years ago with a 95% HPD range of 112,600 to 789,200 years for the divergence between the Clarion Island sample and the North A clade. The estimated divergence time and lower limit of the 95% HPD interval substantially predate not only the establishment of the navy base on Clarion Island but also human colonization of the Americas, estimated sometime after 16,500 years ago (Goebel et al. 2008). Although some recent estimates date humans in North America approximately 23,000 years ago (Holliday et al. 2025), this is still much later than our minimum estimate for the divergence of Spiny‐tailed Iguanas on Clarion Island. However, it is possible that the lineage of iguanas on Clarion Island diverged from their mainland ancestors prior to colonizing the island, either the mainland population was not sampled or became extinct. The former is unlikely given the dense sampling of Zarza et al. (2008; their figure 1), while the latter requires hypothesizing both an unsubstantiated mainland lineage and its extinction; it is more parsimonious to infer that the divergence coincided with colonization. Thus, our results support natural dispersal as the mechanism of colonization. This aligns with studies of other native squamatans on the islands, including Clarion Nightsnakes ( *Hypsiglena unaocularus* ) (Mulcahy et al. 2014), Clarion Whipsnakes ( *Masticophis anthonyi* ) (Myers et al. 2017), and Clarion Tree Lizards ( *Urosaurus clarionensis* ) (Feldman et al. 2011), all of which share origins in mainland northwestern Mexico.

The Clarion Island population of *Ctenosaura* is inferred to be sister to the North A clade of Zarza et al. (2008, 2019), now recognized as *C. brachylopha* (Zarza et al. 2019) (Figure 4). Sequence divergence of the Clarion population from *C. brachylopha* was 0.9%–1.8%, similar to that between the Clarion Nightsnake ( *H. unaocularus* ) and its nearest relative *H. catalinae* (1.2%–1.5%) for the same ND4 marker (Mulcahy et al. 2014). Sequence divergence within *C. brachylopha*, whose distribution extends for hundreds of kilometers along the west coast of Mexico from Sinaloa to Nayarit (Figure 2), was 0.18%–1.43% (Table 1). Sequence divergence between *C. brachylopha* and clades North B, C, and D was 1.96%–3.74%, while that between the Clarion population and clades North B, C, and D was 1.8%–3.4% (Table 1). *Ctenosaura brachylopha* was recently resurrected from a wide‐ranging *C. pectinata* based on mtDNA, microsatellite data, and slight morphological differences (Zarza et al. 2019). *Ctenosaura pectinata* is now considered to occur in southern Mexico from Nayarit on the Pacific Versant to the Isthmus of Tehuantepec (Figure 2) and based on the mtDNA (ND4) data analyzed herein, is paraphyletic with respect to *C. acanthura* , *C. brachylopha*, and the Clarion Island population (Figure 4). At this time, we consider Clarion Spiny‐tailed Iguanas to be an evolutionarily significant unit within *C. brachylopha*, as they are both geographically isolated and genetically distinct from the remaining *C. brachylopha*. More data and analyses are necessary to determine if they warrant separate taxonomic recognition, preferably with more individuals sampled, genomic DNA, and morphological comparisons with populations from mainland Mexico.

Natural dispersal mechanisms, such as rafting on vegetation mats, are well‐documented for iguanas, including the closely related *C. hemilopha* (Davy et al. 2011), other *Ctenosaura* species (Gutsche and Köhler 2008), other species of iguanas (Censky et al. 1998; Rassmann 1997; Scarpetta et al. 2025), and more distantly related lizards (Glor et al. 2005). Iguanas' ability to survive extended periods without food or water and their tolerance to saltwater make them effective colonizers of remote islands (Scarpetta et al. 2025). These traits likely facilitated the arrival of iguanas on Clarion Island, probably floating on vegetation mats originating in the Nayarit–Sinaloa region of mainland Mexico, where several large river drainages discharge into the Gulf of California and Pacific Ocean. The closest known locality of *Ctenosaura brachylopha* to Clarion Island is Zacualpan, Nayarit (Zarza et al. 2019), which is about ~1100 km to the northeast. This should be considered a minimal dispersal distance for the *Ctenosaura* on Clarion, as the tree lizards ( *U. clarionensis* ) and nightsnakes (*H. unaocularus*) on Clarion Island have their closest mainland relatives further north, near the Sonora–Sinaloa border (Feldman et al. 2011 and Mulcahy et al. 2014, respectively), just north of where *C. brachylopha* reaches its northern extent (Zarza et al. 2008). This pattern of dispersal is thought to be caused by surface currents flowing southward down the Gulf of California and a Pacific current flowing westward from the Colima–Guerrero area directly towards the Revillagigedo Archipelago (Kessler 2006). This over‐water dispersal event represents the second largest by any iguana known to date (see Scarpetta et al. 2025); the distance is slightly larger than that between the mainland of Ecuador and the Galapagos Islands, where iguanas are also thought to have dispersed over water (Rassmann 1997).

### 4.2 How Could Iguanas Go Unnoticed for Over 100 Years?

We propose that the dense vegetation and challenging terrain described by early explorers of Clarion may have obscured the presence of iguanas, contributing to their absence from early historical accounts. Townsend (1890) vividly described the conditions on Clarion Island:

The central portion is a plateau about 1000 feet high, with a few elevations perhaps 500 feet higher. It is mostly overgrown with long grass, head high, through which the pedestrian flounders helplessly; the slopes of the lesser elevations are clearer, with scattered bushes and low, scrubby trees. I was not able to reach the plateau until after two hours of laborious struggle through the wilderness of cactus that covers its southern slopes, cutting nearly every yard of the way with a sharp machete. No other members of the party attempted it. Cactus renders all the lower portions of the island practically impassable. (Townsend 1890, 132).

Similar challenges were noted by other early explorers, including Anthony (1898), Hanna (1926), and Beebe (1938). Additionally, many research expeditions to Clarion Island were brief, such as Brattstrom's 1953 visit, which lasted only 3 days (March 23–25) and focused on areas near Sulphur Bay on the southern side of the island (Brattstrom 1955). Later researchers assumed that if iguanas were not observed by Brattstrom and others, they must have been introduced because they are so conspicuous (González‐Sánchez et al. 2021). However, others have noted the wary behavior of the iguanas on Clarion Island, stating they “retreated rapidly on approach, even from a distance” (Scheidt 2017, 123–124). Additionally, Brattstrom also failed to observe Clarion Nightsnakes ( *Hypsiglena unaocularus* ), which were later found to be relatively abundant during surveys in 2013 (Mulcahy et al. 2014) and again in 2023 (J.R.V. & D.I.V.A., personal observation). It is plausible that Brattstrom similarly overlooked the iguanas, given the limited scope and duration of his expedition and the dense vegetation. Consistent and thorough monitoring is needed to understand species composition and interactions in this dynamic archipelago, such as the case for Townsend's Shearwaters, a population of which was also rediscovered in 2012 (Martínez‐Gómez et al. 2015).

Subsequent to the early expeditions, the vegetation of Clarion Island has been significantly altered by invasive species and other disturbances. Following the establishment of a naval outpost in 1979, rabbits, pigs, and sheep were introduced to the island, causing widespread ecological damage (Mendez‐Guardado 2001). Although pigs and sheep were eradicated in the 2000s (Aguirre‐Muñoz et al. 2011), rabbits remain abundant and continue to degrade the native vegetation. A large fire in 1984 further transformed the island's flora, burning the western two‐thirds of the island (Everett 1988). These events have led to the near disappearance of large cactus patches noted by early explorers, which are now rare on the island (personal observations; see Figure 1).

We posit that the iguanas on Clarion Island were historically elusive and that recent alterations to the vegetation have made them more conspicuous. Many species of spiny‐tailed iguanas are difficult to approach in open areas; even the ones on Clarion scurry away into rock crevices or burrows when approached (pers. obs. and Scheidt 2017). Rock outcrops and burrows historically covered by dense cactus and brush could have easily allowed iguanas to avoid detection. The change in habitat may explain why iguanas were not reported by early naturalists and were later assumed to be introduced after the dense vegetation had been cleared by invasive pigs and sheep.

### 4.3 Conservation Implications

The discovery that Spiny‐tailed Iguanas on Clarion Island likely arrived through natural dispersal rather than human introduction has important conservation implications. Unlike invasive species, which often require active management, naturally occurring populations such as that of the Clarion Spiny‐tailed Iguanas represent an integral component of the island's native biodiversity. Because iguanas were previously believed to have been introduced to Clarion, the Mexican government has been trying to eradicate them from the island since 2002 (CONANP 2004; Castillo‐Guerrero et al. 2016). Our research is of critical importance in demonstrating that iguanas are native to Clarion and should be considered part of the natural fauna, which should be conserved rather than eradicated (contra Scheidt 2017). Conservation plans must recognize their ecological role and ensure their protection as a native species and an evolutionarily significant unit (ESU). Understanding natural arrivals to island ecosystems and their consequences for island floral and faunal composition is a challenging area of research (Warren et al. 2015), and the Revillagigedo Archipelago provides an excellent system for exploring this phenomenon.

Protecting the *Ctenosaura* population, along with the island's other native reptile species, should be a priority in conservation planning. Further genetic sampling along the western coast of mainland Mexico could refine our understanding of the population's origins and divergence timelines. Additional genomic data and analyses are necessary to determine the taxonomic status of the *Ctenosaura* population on Clarion Island (here provisionally considered an ESU within *C. brachylopha*) and to assess genetic diversity within the island. Non‐invasive sampling techniques could reveal much about the genomic architecture and health of the population. Addressing the ongoing impacts of invasive species is also essential to ensure the long‐term survival of Clarion's unique fauna and flora. By shedding light on the origin and distribution of *Ctenosaura* on Clarion Island, this study contributes to broader efforts to understand and preserve the biological diversity and ecological integrity of oceanic islands.

## Author Contributions

**Daniel G. Mulcahy:** conceptualization (equal), data curation (lead), formal analysis (lead), methodology (equal), project administration (lead), writing – original draft (equal), writing – review and editing (equal). **Jacobo Reyes‐Velasco:** conceptualization (equal), data curation (equal), investigation (equal), visualization (equal), writing – original draft (equal), writing – review and editing (equal). **Daniel I. Vázquez‐Arce:** conceptualization (supporting), investigation (supporting), writing – review and editing (supporting). **Juan A. Cervantes‐Pasqualli:** investigation (equal). **Juan E. Martínez‐Gómez:** investigation (equal), resources (lead), writing – review and editing (supporting). **Kevin de Queiroz:** conceptualization (equal), methodology (equal), validation (lead), writing – review and editing (equal).

## Conflicts of Interest

The authors declare no conflicts of interest.

## Data Availability

Newly generated sequence data is deposited in GenBank (Accession Number: PV941008).
